# MEMORY CAFÉ PROVIDES INTERGENERATIONAL ENGAGEMENT OPPORTUNITIES FOR LWD CARE-PARTNERS’ LOVED ONES

**DOI:** 10.1093/geroni/igad104.1660

**Published:** 2023-12-21

**Authors:** Janice Whitaker, Diane Berish, Jenny Rank, Meghan McGraw, Erica Husser

**Affiliations:** Nese College of Nursing, Penn State University, State College, Pennsylvania, United States; The Pennsylvania State University, University Park, Pennsylvania, United States; Penn State University, University Park, Pennsylvania, United States; Penn State University, University Park, Pennsylvania, United States; Penn State University, State College, Pennsylvania, United States

## Abstract

To facilitate attendance by care partners attending the Living with Dementia (LWD) education sessions, an optional Memory Café was available at the same location. We describe the design and implementation of the Memory Café, which included evidence-based programming to engage and provide socialization for the older adults. Basic health, safety, person-centered, and care preference information was collected at registration to ensure quality supervision and support during the Memory Café. The Café was facilitated by interprofessional staff and included Opening Minds through Art (OMA), an intergenerational no-fail art-making experience for persons with neurocognitive disorders (“artists”) that fosters creative self-expression and engagement for persons living with dementia (PLWD). Facilitators also integrated reminiscence, music, and movement therapies during the two-hour LWD education session. Nineteen community dwelling PLWD attended an average of two of the four Memory Café sessions, 26 participants were recruited from a care home at one location, and 124 volunteers were paired with PLWD for OMA and attended at least one Memory Café (volunteers included undergraduate nursing students, graduate students, Penn State faculty and staff, and community members). Fifty-one pre- and post-mood assessments were completed by the 19 community dwelling OMA artists (using a 5-point Likert scale of faces, from frowning to smiling, to indicate how they felt in the moment) and data showed mood improved by an average of .67 points (standard deviation = .95). Care partner evaluation data was positive; 8 (22%) reported that having the Memory Café option made it possible for them to attend LWD.

